# A Blooming Season for Natural Polymers and Biopolymers

**DOI:** 10.3390/molecules28073207

**Published:** 2023-04-04

**Authors:** Sylvain Caillol

**Affiliations:** ICGM, University of Montpellier, CNRS, ENSCM, 34000 Montpellier, France; sylvain.caillol@enscm.fr

The year 2023 is particularly remarkable because we are celebrating the 25th anniversary of the 12 principles of Green Chemistry described in the groundbreaking book *Green Chemistry: Theory and Practice* co-authored by Paul Anastas and John C. Warner and published in 1998. The use of renewable resources is one of the most important of these principles. This approach is in particular very important for polymers which find very varied industrial applications. Hence, the past 25 years have seen a booming number of articles and reviews describing the use of bio-resources as a starting point for original polymer chemistry. Indeed, the use of renewable resources could help the chemical industry to answer to some of the current challenges of our society, including development facing global warming and limited fossil resources. Hence, the latest developments not only have created a library of polymeric materials exhibiting a wide range of properties to fulfill the requirements of various industrial applications, but also have improved our knowledge and understanding of the structure and reactivity of the complex biomass. Additionally, these biopolymers could allow addressing unmet needs and obtaining new properties that cannot be achieved with petroleum-based chemicals. They could also help avoiding the use of harmful substances, thus contributing to restoring the chemical industry’s sustainability.

This Special Issue on "Natural Polymers and Biopolymers" is prompted by the increasing attention that the field of “green polymers” is receiving. It presents cutting-edge research works focusing on the use of bio-resources for polymeric materials and shows how natural polymers and biopolymers, with their interesting and original properties, are destined to replace and outperform oil-based polymers.

The first article is a perspective paper dedicated to the evaluation of production of sustainable production of polyhydroxyalkanoates (PHAs), which remain promising candidates for commodity bioplastic production [1]. This article focused on defining obstacles and solutions to overcome cost performance metrics that are not sufficiently competitive with current commodity thermoplastics. To that end, this review described various process innovations that build on fed-batch and semi-continuous modes of operation as well as methods that lead to high-cell-density cultivations. Finally, future directions for efficient PHA production and relevant structural variations are discussed.

The second paper is a perspective paper on the synthesis and applications of a graphene quantum dots–nanocellulose Composite. Graphene quantum dots (GQDs) are zero-dimensional carbon-based materials, while nanocellulose is a nanomaterial that can be derived from naturally occurring cellulose polymers or renewable biomass resources [2]. The unique geometrical, biocompatible, and biodegradable properties of both these remarkable nanomaterials have caught the attention of the scientific community in terms of fundamental research aimed at advancing technology. This study reviewed the preparation, marriage chemistry, and applications of GQDs–nanocellulose composites and unlocks windows of research opportunities for GQDs–nanocellulose composites.

The purpose of third paper, a review on locust bean gum, a vegetable hydrocolloid, was to report the structural characteristics of locust bean gum, its biosynthetic origin and its chemical isolation, and its applications and derivatives either by functionalization or cross-linking [3].

The fourth paper considered new bio-based printable materials [4]. In this work, bio-based poly(trimethylene terephthalate) (PTT) blends were combined with pyrolyzed miscanthus biocarbon to create sustainable and novel filaments for extrusion 3D printing.

The aim of the fifth paper was to carefully investigate the stability of acetal-containing monomers, mainly focusing on reaction conditions during melt polycondensation [5]. Hence, it allows shedding new light on the underlying mechanism governing the observed behavior, thus aiding the development of solvent-free experiments, and later, material design.

Furfuryl alcohol is a promising bio-based furan derivative. The objective of the sixth paper was to study the control of ring opening polymerization of furfuryl alcohol according to different parameters in order to open the way to various applications [6].

Adhesion onto polypropylene (PP) and poly(ethylene terephthalate) (PET) is a challenge. The seventh paper successfully addressed this challenge with new emulsion polymers based on vegetable oil monomers [7]. In this study, the best-performing latex adhesives containing up to 45 wt. % of high-oleic soybean-oil-based monomer fragments demonstrated promising efficiency in the testing of PET to PP and coated-to-uncoated paperboard substrate pairs, resulting in substrate failure during the adhesive testing.

The eighth paper interestingly compared the effect of native starch and sugarcane bagasse fibers for the preparation of biocomposites with natural reinforcements [8]. It demonstrated that although the environmental benefit of the prepared biocomposites is similar, the overall performance of the bagasse-fiber-reinforced polylactide (PLA) composites is better than that offered by the PLA/starch composites.

The ninth paper focused on the chiral resolving ability of the commercially available amylose (3,5-dimethylphenylcarbamate)-based chiral stationary phase (CSP) toward four chiral probes representative of four kinds of stereogenicity [9]. This study confirmed that the use of the amylose-based CSP in HPLC is an effective strategy for obtaining the resolution of chiral compounds containing any kind of stereogenic element

The indiscriminate use of plastic in food packaging contributes significantly to environmental pollution, promoting the search for more eco-friendly alternatives for the food industry. The tenth paper studied five formulations of biodegradable cassava starch/gelatin films [10]. In the outcome of this study, an optimal formulation was obtained to develop cassava starch/gelatin-based films in a 53/47 ratio, plasticized with glycerol using the casting method that would meet the expectations of the model polyethylene film for food-packaging applications

Biopolymers, especially polysaccharides (e.g., gum arabic), are widely applied as drug carriers in drug delivery systems due to their advantages. Curcumin, with high antioxidant ability but limited solubility and bioavailability in the body, can be encapsulated in gum arabic to improve its solubility and bioavailability. The eleventh paper studied the released of curcumin in various conditions [11].

The aim of the twelfth paper was to experimentally and numerically optimize the process of the sulfation of ethanol lignin birch wood with a mixture of sulfamic acid and urea in a 1,4-dioxane medium and to characterize the structure and thermochemical properties of the sulfated ethanol lignin [12]. Using experimental and computational methods, the optimal conditions for the process of birch ethanol lignin sulfation with a sulfamic acid–urea mixture to provide a high yield of sulfated product (more than 96 wt. %) with a sulfur content of 8.1 wt. % were established.

Despite a number of studies addressing the issues of low deformation and the poor impact resistance of PLA blends without sacrificing stiffness and strength, it is still not clear how the phase structure and phase interaction in multicomponent blends contribute to the impact modification of ternary blends. The objective of the thirteenth article was to report on the effect of adding an intermediate elastomer phase and the blend composition on the morphology development of fully bio-based PLA-Polyamide (PA) blends prepared by melt blending [13].

Starch is a macroconstituent of many foods and 40% of starch is used in nonfood industries as an additive in cement, paper, gypsum, adhesives, bioplastics, composites, etc. In particleboards, they are used as a substitute for binders such as urea-formaldehyde, phenol–formaldehyde, and other petroleum derivatives. The aim of the fourteenth article was to study the manufacturability of particleboards made from giant reed with gypsum plaster and starch following a method based on the wood industry dry process but with variations so that it can be produced in the particleboard industry [14].

Taken together, the articles in this issue reveal not only the growing attention paid to the use of renewable resources and the substitution of petroleum-based substances, following the trends in society, but also all the interest and promises of this chemistry for the improvement of current materials and the design of new sustainable materials.
